# Sheep’s Head as an Anatomic Model for Basic Training in Endoscopic Sinus Surgery

**DOI:** 10.3390/medicina59101792

**Published:** 2023-10-09

**Authors:** Constantin Stan, Laszlo Peter Ujvary, Cristina Maria Blebea, Doiniţa Vesa, Mihai Ionuţ Tănase, Mara Tănase, Septimiu Sever Pop, Doinel Gheorghe Rădeanu, Alma Aurelia Maniu, Marcel Cosgarea

**Affiliations:** 1 Department of ENT, “Iuliu Haţieganu” University of Medicine and Pharmacy, 400012 Cluj-Napoca, Romania; const.stan@ugal.ro (C.S.); cristina_blebea@yahoo.com (C.M.B.); dr.mihaitanase@gmail.com (M.I.T.); marabulmaci@yahoo.com (M.T.); severpop@me.com (S.S.P.); dr.radeanu@gmail.com (D.G.R.); almacjro@yahoo.com (A.A.M.); rcosgarea@yahoo.com (M.C.); 2 Department of Surgical Clinic, Faculty of Medicine and Pharmacy, “Dunărea de Jos” University, 800008 Galați, Romania

**Keywords:** surgical training, simulation, sinus surgery, animal model, FESS

## Abstract

*Background and Objectives*: This study aims to establish the sheep head as a viable anatomical model for training in functional endoscopic sinus surgery through comprehensive anatomical examination and training-based assessment of participants’ satisfaction. *Materials and Methods*: Participants were divided into three groups according to their prior experience in endoscopic sinus surgery; in total, 24 participants were included. Each participant in the study was assigned to perform the designated procedures on a single sheep’s head. Following the completion of the procedures, each participant was provided with a 14-item comprehensive satisfaction questionnaire with a scale attributed from 1 to 5. The normality of distribution was checked by applying the Shapiro-Wilk Test. The Kruskal–Wallis test was applied to compare study group sentiment of agreement towards individual procedures. *Results*: No significant differences were noted between the answers of the different groups. For the resident group, the average satisfaction score was 4.09 ± 0.54; junior specialist group 4.00 ± 0.55; for the senior specialist group overall satisfaction average score was 4.2 ± 0.77. *Conclusions*: The sheep’s head can be successfully used for learning and practicing manual skills and the use of instruments specific to functional endoscopic sinus surgery. Moreover, the sheep head model can be used for training in other diagnostic or surgical procedures in the field of otorhinolaryngology, such as endoscopy of the salivary glands, open laryngotracheal surgery, or in otologic surgery, but also in other different surgical fields such as neurosurgery, ophthalmology or plastic surgery. Despite the differences between the ovine model and human anatomy, it provides a resourceful and cost-effective model for beginners in endoscopic nasal surgery.

## 1. Introduction

The process of surgical training in Functional Endoscopic Sinus Surgery (FESS) heavily relies on the availability and accessibility of well-equipped laboratories that facilitate endoscopic dissection on cadaveric heads. However, the limited availability of cadavers due to medical, ethical, and moral considerations has led to the exploration of alternative methods, specifically the utilization of anatomical models that closely resemble the human head [1]. In order to achieve this objective, efforts have been made to develop techniques that can ease the learning process. One proposed approach involves using sheep heads as anatomical ex vivo models, although the literature on this subject is currently scarce and lacks comprehensive studies [2].

For endoscopic sinus surgery training, both the in-vivo ovine model and cadaveric simulations offer high handling fidelity. In certain countries, in-vivo animal simulation is prohibited, making animal cadaveric simulation a more accessible substitute [3,4]. The ovine sinus model allows the acquisition of endoscopic surgical skills using video endoscopic techniques. It is cost-effective, portable, and adaptable for use in various settings [5]. Moreover, the ex-vivo ovine model for training does not require institutional review committee approval and raises fewer ethical concerns compared to live animal models [5,6].

This study aims to establish the sheep head as a viable anatomical model for training in functional endoscopic sinus surgery through comprehensive anatomical examination and training-based assessment of participants’ satisfaction. 

## 2. Materials and Methods

### 2.1. Image Acquisition for Anatomical Description and Anatomical Sectioning

After ethical approval was granted (AV258/28.02.2022), an anatomical feasibility study was undertaken regarding the sheep head as an alternative for FESS training. For the anatomical study, 26 fresh-frozen adult sheep heads (Native Romanian Turcana sheep) were procured from the local slaughterhouse (veterinary clearance was obtained), at a price of 10 Romanian lei (RON) a piece, roughly the equivalent of two euros a piece. One head was defrosted and used as a sample for imaging acquisition and endoscopy and the other head was used to provide sectional anatomical details. The second head (frozen at −20 °C) was serially sectioned in sagittal and transverse planes. The sagittal plane was paramedian to the septum, dividing the skull into two equal parts. The transverse sections were 2.5 cm in thickness. Sectional photographs were taken, and important anatomical elements were digitally labeled. The obtained information was presented to the participants through oral presentations and written handouts. 

### 2.2. Model Preparation for Surgery and Instrumentation

Prior to the initiation of the maneuvers, a preparatory phase was undertaken, during which the fresh frozen sheep heads were allowed to undergo a gradual thawing process at room temperature for a duration of approximately 14 h. As a subsequent step in the preparatory process, the nasal cavities of the sheep heads were irrigated with a saline solution. Ultimately, before beginning the evaluation process, the tip of the nose was sectioned to facilitate endoscopic maneuvering, a useful aspect also presented by other authors [7]. Table 1 lists the equipment and instruments used for training.

**Figure 1:**
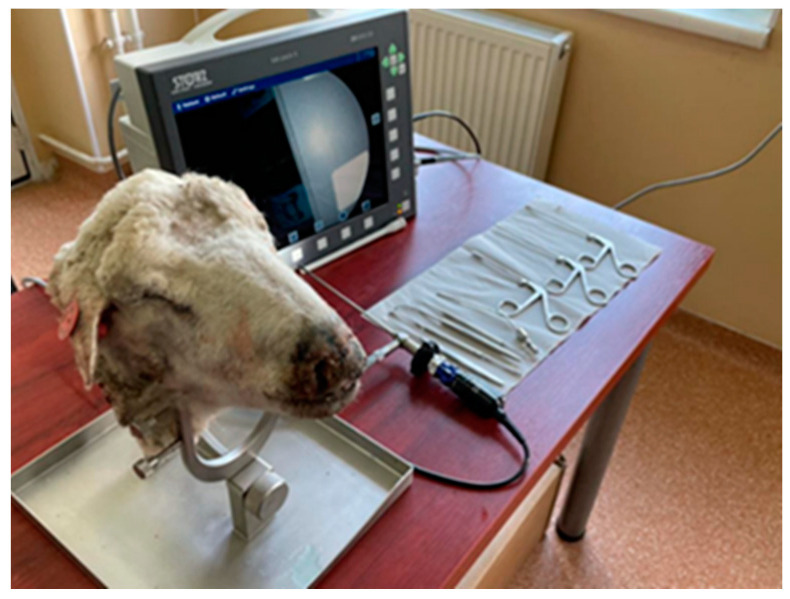
View of the sheep’s head secured in the Karl Storz^®^ special head holder.

The support was innovated by Mladina and Karl Storz Gmbh to fix in position and secure the lamb’s head (Figure 1) [7,8]. In our study, we used it for the sheep’s head, which, although it is more voluminous, could be fixed without problems.

A series of basic endoscopic maneuvers using the sheep’s head as a model was performed by 24 participants. Each participant was allocated one sheep head (Table 2).

### 2.3. Participant Selection

Participants were divided into three groups according to their prior experience in endoscopic sinus surgery. The first group was formed by 10 first-year otolaryngology resident doctors with minimal or no experience in endoscopic sinus surgery. The second group consisted of 10 junior otolaryngology specialists with 3–5 years of exposure to rhinology procedures. The third group was formed by four senior otolaryngologists with over 20 years of experience. 

### 2.4. Participant Assessment—Satisfaction Questionnaire

Each participant in the study was assigned to perform the designated procedures on a single sheep’s head, targeting both nasal fossae. Following the completion of the procedures, each participant was provided with a 14-item comprehensive satisfaction questionnaire (File S1). For each answer, a scale from 1–5 was attributed, 1 denoting “totally disagree”, and 5 signifying “completely agree”. This allowed participants to provide nuanced feedback, enabling a comprehensive assessment of their overall satisfaction and perception of the performed procedures. 

### 2.5. Statistical Analysis

For statistical analysis, Statistical Package for the Social Sciences (SPSS) 26.0 (Armonk, NY, USA: IBM Corp) was used. Data from the questionnaire was collected as numerical variables (1–5). For each group, mean and standard deviation were calculated for an individual answer within the questionnaire. The normality of distribution was checked by applying the Shapiro–Wilk Test. The Kruskal–Wallis test was applied to compare study group sentiment of agreement towards individual procedures.

## 3. Results

### 3.1. Basic Endoscopic Anatomy

**Figure 2:**
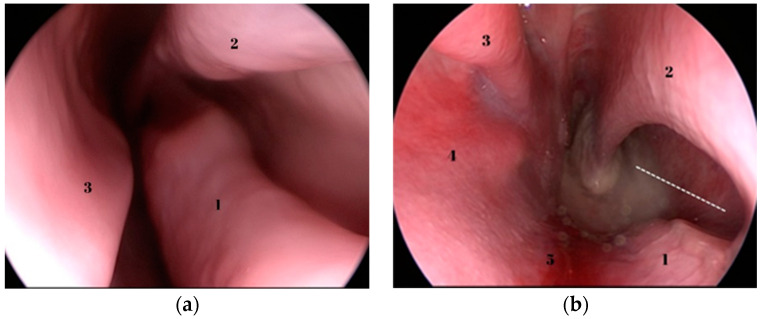
(**a**) Endoscopic view of the sheep’s left nasal fossa. 1—inferior part of inferior turbinate; 2—superior part of inferior turbinate; 3—nasal septum; (**b**) endoscopic view of the posterior septal defect specific to the ovine model described by Mladina [7]—dotted white line. Right nasal fossa, 1—the lower limit of the septal defect; 2—the superior limit of the septal defect; 3—inferior turbinate; 4—lateral nasal wall; 5—nasal floor.

**Figure 3:**
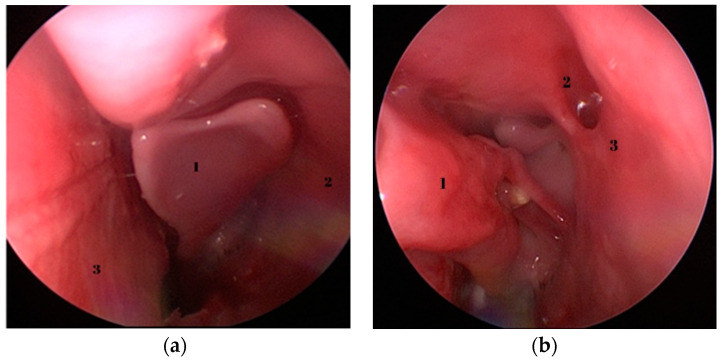
(**a**) Endoscopic view—left nasal fossa—1—middle turbinate; 2—lateral nasal wall; 3—septum. (**b**) Endoscopic view—left nasal fossa; 1—medialized middle turbinate; 2—natural ostium of the maxillary sinus; 3—uncinate process.

**Figure 4:**
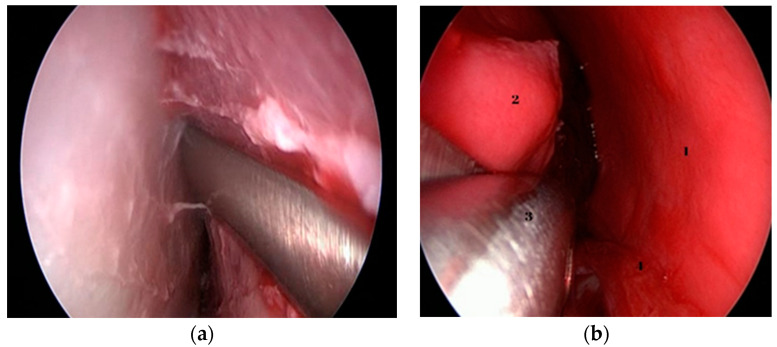
Endoscopic images of sub-perichondral dissection of the septum and turbinate fracture. (**a**) Sub-perichondral dissection of the septum, (**b**) Instrumental lateral fracture of the inferior turbinate. 1—lateral nasal wall; 2—inferior turbinate; 3—Cottle dorsal scissors; 4—insertion of the inferior turbinate to the lateral nasal wall.

Relevant anatomical structures regarding the endoscopic dissection can be seen in Figure 2, Figure 3 and Figure 4. Opposed to humans, the inferior turbinate is comprised of two parts.

The excision of the inferior turbinate permits the visualization of the middle turbinate. By gently and medially displacing it using an elevator, the natural ostium of the maxillary sinus becomes visible. To further enhance access and facilitate surgical intervention, the backbiter antrum punch can be employed to enlarge the natural ostium, resulting in the creation of a broad middle antrostomy.

### 3.2. Procedures and Evaluation

Following the extraction of a foreign body, attention was directed to raising the mucosal flap, bone resection and repositioning of the mucosal flap to mimic the maneuver performed during an actual turbinoplasty. Endoscopic septoplasty was undertaken as shown in Figure 4a. Following the initial procedures, the inferior turbinate was resected as a whole to facilitate the visualization of the middle turbinate—Figure 4b.

**Figure 5:**
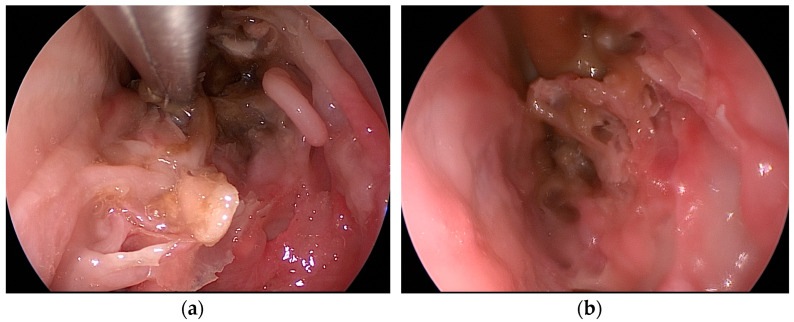
(**a**,**b**) Endoscopic images of instrumental removal of ethmoidal cells.

By medializing the middle turbinate, the natural ostium of the maxillary sinus is also pointed out, which was widened with the help of the backbiter antrum punch to create an anthrostomy. The last procedure undertaken was the ethmoidectomy—Figure 5a,b.

The assessment of the overall satisfaction and perception of the performed procedures was as follows in Table 3.

**Figure 6:**
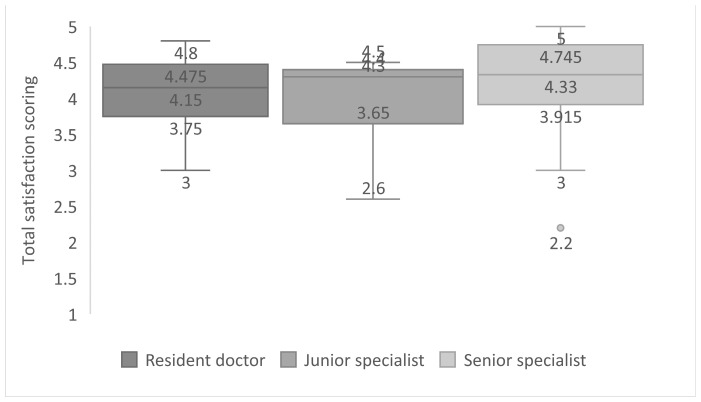
Total satisfaction scoring regarding participant groups.

For the resident group, the average satisfaction score was 4.09 ± 0.54; junior specialist group 4.00 ± 0.55; for the senior specialist group overall satisfaction average score was 4.2 ± 0.77 (Figure 6). Considering the average score for all responses noted for each individual group, no statistical differences can be noted, with a *p*-value of 0.598.

## 4. Discussion

Overall satisfaction in all groups was equal to or above 4 out of 5, meaning that a strong agreement has been reached in all groups, regardless of previous experience, in favor of considering the sheep’s head as a useful model for endoscopic endonasal surgical training.

The extraction of a foreign body from the nasal fossae necessitates hand–eye coordination, particularly due to the utilization of a 2D endoscopy image, which requires accurate depth perception and spatial orientation. Participants in all groups agreed that simulating foreign body extraction is a good procedure to learn utilizing this model. 

Although other authors [7,8] recommend the complete removal of the anterior septal portion for better endoscopic visualization, we chose to keep a part of the anterior septum, considering the advantage it offers us for practicing septoplasty. Even so, this specific modification was advantageous in facilitating the accessibility and maneuverability required during endoscopic procedures. All study group participants were in strong agreement with the benefits offered by the sheep model in endoscopic septal resection training, with no statistical differences noted between groups. 

The training scenario simulating a turbinoplasty provided a learning experience to enhance participants’ endonasal tissue handling skills. This exercise proved to be particularly advantageous for beginners, as it offered a controlled environment in which to refine their skills.

The deliberate and controlled execution of partial resection and mucosal flap manipulation within the nasal cavity proved to be an invaluable training tool. By carefully excising a segment of the inferior turbinate, it becomes possible to accentuate the prominence of the middle turbinate, which serves to identify the natural ostium of the maxillary sinus.

Although the overall satisfaction was high in all groups there are a few drawbacks when considering the anatomical similarities. Similarity of anatomical landmarks compared to humans received an average score of 3.1. Also, procedures like ethmoidectomy and maxillary anthrostomy did not receive an overall high satisfaction score, averaging 2.6 for ethmoidectomy and 3.6 for anthrostomy. 

There are few studies that have highlighted the utility of the ovine model as a viable substitute for surgical training in the field of functional endoscopic sinus surgery.

Gardiner [9] was the first author to propose the sheep’s head for training in endoscopic sinus surgery, proposing both simple and more complex maneuvers. Awad et al. [5,6] also proposed the sheep’s head for surgical training in endoscopic rhino-sinusal surgery, showing clear advantages of its use, demonstrating the face, content and construct validation of this anatomic model. Mladina et al. [7,8,10] in their studies chose the lamb’s head, due to its smaller size, which allows the use of standard endoscopic rhino-sinusal surgery instruments. He implemented a training program in endoscopic rhino-sinusal surgery using the lamb’s head, which proved to be useful for the trainees and allowed the transition to the next level in endoscopic sinus surgery training, i.e., human cadaver training. 

Although 3D printing technology has been frequently used for preoperative assessment in endoscopic sinus surgery [11], it is also used to create anatomical models for surgical training in FESS, with the possibility of creating high-fidelity models, being able to simulate certain sinus pathologies, benefiting from validation in this sense, but without benefiting from the real feeling of the tissues, an important disadvantage compared to the sheep’s head training.

Even with the mentioned drawbacks, we consider that the ovine FESS model can be an integrative part of surgical training alongside artificial models. Combining the precise anatomy of the artificial models with the feel and overall experience of the ovine model, a more complex teaching experience could be achieved, considering the importance of functional endoscopic sinus surgery [12].

Otologic surgery is another field where the ovine model has been successfully implemented in the training and preparation of young doctors. Anschuetz et al. [13] carried out a study that developed and validated the ovine model for surgical training in endoscopic ear surgery. The anatomy of the sheep’s ear was compared with that of humans and was used to perform a series of otological surgical procedures such as canaloplasty, myringoplasty, and ossiculoplasty. Procedures were subjectively evaluated using a postoperative questionnaire, using a scale from 1 to 10. Mean procedure times were as follows: canaloplasty (29.7 ± 13.2 min), middle ear dissection (7.7 ± 2.6 min), myringoplasty (7.7 ± 4.3 min), and ossiculoplasty (10.4 ± 2.7 min). Canaloplasty and flap elevation time decreased from 46.4 to 16.2 min over the study (absolute difference: 30.2 min, 95% CI 22.28–38.12). Subjective ratings were high: tissue quality (8.9/10), overall satisfaction (8.3), and learning experience (8.8). Beckmann et al. [14] carried out a study in which, using the ovine model, they evaluated the learning curve of laser stapedotomy for trainees, with considerable improvement of operative times once the maneuver is repeated. The experienced surgeon maintained a steady average time of 15:01 min throughout the training without any intraoperative issues. The fellow reduced surgical time gradually from 27:21 (first five cases) to 24:10 min (last five cases), and the resident also reduced this time from 42:38 to 21:08 min. Training methods in otosclerosis surgery are very limited, which is why the ovine model proved to be more than useful for this purpose.

Fernandez et al. [15] using eight lamb heads simulated rare situations that may occur intraoperatively in stapes surgery. Thus, floating footplate, footplate fracture, luxation of the incus or necrosis of the long process, overhanging facial nerve, and obliterative otosclerosis could be simulated. A subjective questionnaire with a scale from 1 to 10 was used to determine the satisfaction of the study participants. Taking into account that the options for surgical training in stapes surgery are very limited, the ovine model used in this regard proved to be an excellent learning method for young otologists.

The sheep model was also used for the training of plastic surgery fellows. Isaacson et al. [16] evaluated the possibility of surgical training using the sheep’s head for a series of plastic surgery techniques performed for pathologies of the eyelids and the orbit. They harvested 10 sheep heads after the completion of an in vivo study. They evaluated a series of procedures such as upper eyelid blepharoplasty, ptosis repair, upper eyelid repair procedure in facial paralysis, lateral canthotomy, lower acantholysis, narrowing of the lower eyelid, and transconjunctival approach to the floor of the orbit. The overall utility was excellent but remarks were made for limitations due to anatomical variations, one of which is the anatomy of the lacrimal system. 

In the field of laryngology the ovine model was used for training in open laryngotracheal surgery. The head and neck were used to simulate a series of surgical procedures, such as tracheotomy, laryngoplasty, tracheal resection with tracheal sutures, and laryngectomy with pharyngeal sutures. This time, the ovine model could be successfully implemented for the training of open surgical techniques, having the advantage of the learning of surgical skills and tissue handling on a model close to humans [17].

The ovine model was also described in the field of dentistry to aid in the learning curve of sinus augmentation. Valboneti et al. analyzed the sheep and human maxillary sinuses anatomy using cone beam computed tomography and histology. Obvious maxillary sinus differences were identified between the human head and ovine model, which were taken into account in order to carry out the experimental procedures [18].

The ovine model was also successfully used for training in sialendoscopy, Borner et al. [19] implemented such a training program in salivary gland endoscopy using the sheep’s head, a novelty in the field considering that, for training in these procedures, pig’s head was used for several years. The Stenson and Wharton ducts could be successfully visualized endoscopically and therapeutic maneuvers were also simulated by removing a stone from the Stenson duct.

The ovine head was also frequently used for training in different neurosurgical procedures. Kamp et al. [20] simulated a brain tumor model using agar-agar solution injected into fresh sheep brains. The tumor model would vary depending on the concentration of the injected solution, so a high concentration could cause the appearance of well-defined tissue masses, simulating possible brain metastases, while a solution with a lower concentration could cause the appearance of a diffuse infiltrative tissue, thus simulating a primary tumor formation at the level of the brain tissue. This model made it possible to simulate different intraoperative scenarios for young neurosurgeons, with the advantage of learning manual skills and obtaining a haptic sense, in a much more relaxed environment and without the pressure of real intraoperative scenarios.

Korotkov et al. [21] looked for an accessible anatomical model for the training of anterior clinoidectomy, the procedure performed in the case of vascular and tumoral conditions located at the frontotemporal level, which requires a high level of surgical training. Five formalized sheep heads were evaluated, at the level of which, through silicone injection, the presence of dural and extradural lesions was simulated. All the procedures were carried out successfully, thus demonstrating the fact that the sheep head model can be a good alternative to the human cadaver.

Another use of the sheep head model belongs to Altunrende et al. [22], who used the sheep skull for the training of neurosurgeons and ophthalmology residents in performing orbitotomy and frontal craniotomy using dissection under the microscope with good results and with satisfaction in the training of microsurgical techniques for the approach of the optic nerve and optic canal.

Limitations of this study include a small number of participants who belonged to a single training institution. Moreover, our study did not evaluate the transfer of skills acquired using this anatomical model in real endoscopic sinus surgery. Although effective in preparation, the ovine model has a major disadvantage compared to medical practice due to the lack of vascularization and the lack of inflammatory reaction of the rhino-sinusal mucosa often encountered in surgical practice.

In this sense, to extend our research and to eliminate this limitation, in vivo training sessions for endoscopic sinus surgery could be performed using sheep under general anesthesia with orotracheal intubation. In this way, participants can also face intraoperative bleeding, an essential aspect of the surgical training of young doctors. After performing the surgical procedures, in order to avoid their possible suffering, the sheep would be euthanized in accordance with ethical aspects.

At the moment, the training of young doctors in endoscopic sinus surgery is done through the residency program, which involves practicing directly on the live patient, in the operating room under the direct supervision of an experienced guiding surgeon. However, this determines the limitation of the surgical gestures that can be performed by the young doctor, to avoid possible intra- and post-operative complications. Regarding surgical training in FESS using alternative anatomical models, there is still no organized framework in many countries, leaving room to extend the solutions to this particular ovine model too. To the best of our knowledge, there are various courses organized in university centers in our country, on synthetic anatomical models or even on the ovine model, as is otherwise organized in our center. In this sense, we want to improve the simulation conditions by using in the future the in vivo ovine model, which comes with a series of clear advantages.

## 5. Conclusions

The sheep’s head can be successfully used for learning and practicing manual skills in the use of instruments specific to functional endoscopic sinus surgery, but also for learning some basic endoscopic sinus procedures. Moreover, the sheep head model can be used for training in other diagnostic or surgical procedures in the field of otorhinolaryngology, such as endoscopy of the salivary glands, open laryngotracheal surgery, or in otologic surgery, but also in other different surgical fields, such as neurosurgery, ophthalmology or plastic surgery. Despite the differences between the ovine model and human rhino-inusal anatomy, it provides resources and minimal cost for beginners who want to develop skills applicable to their practice accuracy.

Similar data from the literature are quite limited, which is why this study should have an important role in the implementation of an anatomical model of a sheep’s head for the training in functional endoscopic sinus surgery in our training center and others, considering the accessibility, reliability, and viability of the sheep’s head and very good results obtained from its use.

## Figures and Tables

**Table 1:** Equipment and instruments used for endoscopic maneuvers.

Nr	Equipment (Germany—KARL STORZ SE & Co. KG, Tuttlingen)
1.	Karl Storz TelePack X
2.	Karl Storz rigid Hopkins telescopes 0° and 30°
3.	Mladina head holder (Karl Storz^®^)
4.	Blakesley nasal forceps (Karl Storz^®^)
5.	Backbiter antrum punch (Karl Storz^®^)
6.	Cottle dorsal scissors (Karl Storz^®^)
7.	Freer elevator (Karl Storz^®^)
8.	Sickle knife (Karl Storz^®^)
9.	Straight and curved curette (Karl Storz^®^)
10.	Frazier suction tube (Karl Storz^®^)
11.	Scalpel handle with no. 11 blade (Karl Storz^®^)

**Table 2:** Surgical steps undertaken by participants.

Nr	Surgical Step
1.	Foreign body removal (round plastic, popcorn)
2.	Endoscopic septal resection
3.	Endoscopic turbinoplasty
4.	Maxillary anthrostomy
5.	Ethmoidectomy

**Table 3:** Satisfaction questionnaire assessment.

Questions	ENT Resident Mean (SD)	ENT Junior Mean (SD)	ENT Senior Mean (SD)	*p* Value ^1^
The similarity of anatomical structures to humans	3.1 (0.7)	3.2 (0.6)	3.0 (0)	0.943
Realistic perception of the mucosa	4.7 (0.45)	4.4 (0.8)	4.33 (0.47)	0.386
Realistic perception of bone tissue	4.4 (0.66)	4.1 (0.83)	4 (0)	0.486
Good depth perception	4.2 (0.74)	4.4 (0.48)	5 (0)	0.391
Good applicability of the basic FESS instruments	4.1 (0.94)	4.4 (0.66)	4 (0)	0.475
Useful to improve hand-eye coordination	4.2 (0.6)	4.4 (0.48)	5 (0)	0.660
Useful to improve surgical technique	4.1 (0.83)	3.6 (0.66)	4.33 (0.47)	0.317
Generally useful for basic endoscopic sinus surgery training	4.1 (0.53)	3.8 (0.6)	4.33 (0.47)	0.130
Useful for endoscopic examination of the nasal cavities	4.4 (0.48)	4.5 (0.5)	5 (0)	0.340
Useful for extraction of a foreign body	4.8 (0.4)	4.5 (0.5)	4.66 (0.47)	0.511
Useful for the maxillary antrostomy	3.6 (0.8)	3.6 (0.48)	3.66 (0.47)	0.342
Useful for the ethmoidectomy	3 (0.73)	2.6 (0.66)	2.2 (0.4)	0.191
Useful for the septoplasty	4.8 (0.4)	4.2 (0.4)	4.66 (0.47)	0.582
Useful for the lower turbinoplasty	3.8 (0.4)	4.4 (0.48)	4.66 (0.47)	0.088

^1^ = Kruskal-Wallis test; ENT = otolaryngology; SD = standard deviation; Values for each question and for each group are presented as mean and standard deviation. For most of the answers, no significant difference were noted between the answers of the different groups.

## Data Availability

The data presented in this study are available on request from the corresponding author. The data are not publicly available due to privacy and ethical reasons.

## Supplementary Materials

The following supporting information can be downloaded at: https://www.mdpi.com/article/10.3390/medicina59101792/s1, File S1. Satisfaction Questionnaire.
