# Supplementary material for: Sheep’s Head as an Anatomic Model for Basic Training in Endoscopic Sinus Surgery
Source: Medicina (Kaunas). 2023 Oct 9;59(10):1792. doi: 10.3390/medicina59101792 (PMC10608182; doi:10.3390/medicina59101792)
Supplement: Supplementary file 1 [file medicina-59-01792-s001.zip › medicina-2630695-supplementary.pdf]

NAME INITIALS___________________________

DATE___________

Level of experience in endoscopic sinus surgery:

- first-year otolaryngology resident doctor ☐

- junior otolaryngology specialist ☐

- senior otolaryngologist ☐

	**Totally disagree**	**Disagree**	**Undecided**	**Agree**	**Completely agree**
**The similarity of anatomical structures to humans**	1	2	3	4	5
**Realistic perception of the mucosa**	1	2	3	4	5
**Realistic perception of the bone tissue**	1	2	3	4	5
**Good perception in depth**	1	2	3	4	5
**Good applicability of the basic instruments in FESS**	1	2	3	4	5
**Useful to improve hand-eye coordination**	1	2	3	4	5
**Useful to improve surgical technique**	1	2	3	4	5

**Generally useful for basic endoscopic sinus surgery training**	1	2	3	4	5
**Useful for endoscopic examination of the nasal cavities**	1	2	3	4	5
**Useful for extraction of a foreign body**	1	2	3	4	5
**Useful for the maxillary antrostomy**	1	2	3	4	5
**Useful for the ethmoidectomy**	1	2	3	4	5
**Useful for the septoplasty**	1	2	3	4	5
**Useful for the lower turbinoplasty**	1	2	3	4	5

TOTAL SCORE:
